# High Prevalence and Genotype Diversity of Anal HPV Infection among MSM in Northern Thailand

**DOI:** 10.1371/journal.pone.0124499

**Published:** 2015-05-01

**Authors:** Taweewat Supindham, Suwat Chariyalertsak, Utaiwan Utaipat, Toshiyuki Miura, Darin Ruanpeng, Nuntisa Chotirosniramit, Natthapol Kosashunhanan, Patcharaphan Sugandhavesa, Pongpun Saokhieo, Radchanok Songsupa, Sumalee Siriaunkgul, Antika Wongthanee

**Affiliations:** 1 Research Institute for Health Sciences, Chiang Mai University, Chiang Mai, Thailand; 2 Department of Clinical Medicine, Institute of Tropical Medicine, Nagasaki University, Nagasaki, Japan; 3 Depart of Pathology, Faculty of Medicine, Chiang Mai University, Chiang Mai, Thailand; Asociacion Civil Impacta Salud y Educacion, PERU

## Abstract

**Background:**

HPV infection is common and may cause cancer among men who have sex with men (MSM). Anal HPV infection (HPV+) was found in 85% of HIV-positive (HIV+) and 59% of HIV-negative (HIV-) MSM in Bangkok, central Thailand. As little is known about HPV in this group in northern Thailand, we studied MSM subgroups comprised of gay men (GM), bisexual men (BM), and transgender women (TGW).

**Methods:**

From July 2012 through January 2013, 85 (42.5% of 200) GM, 30 (15%) BM, and 85 (42.5%) TGW who practiced receptive anal intercourse were recruited after informed consent, followed by self-assisted computer interview, HIV testing, and anal swabs for HPV genotyping.

**Results:**

Of 197 adequate specimens, the overall prevalence of any HPV was 157 (80%). Prevalence was 89% (76/85) in GM, 48% (14/29) in BM, and 81% (67/83) in TGW. The most common high-risk types were HPV16 (27% of 197), HPV58 (23%), and HPV51 (18%). Prevalence of high-risk types was 74% in 85 GM, 35% in 29 BM, and 71% in 83 TGW. Prevalence of any HPV type, or high-risk type, was 100% and 94%, respectively, among 48 HIV+ MSM, 70% and 54% among 120 HIV- MSM. Of the 197 specimens, 36% (70) had HPV types 16 and/or 18 in the bivalent vaccine, compared to 48% (95) with ≥1 of types 16/18/06/11 in the quadrivalent, 56% (111) for 16/18/31/33/45/52/58 in the 7-valent, and 64% (126) for 16/18/31/33/45/52/58/06/11 in the 9-valent. HIV+, GM, and TGW were independently associated with HPV infection.

**Conclusions:**

We found higher rates of both any HPV and high-risk types than previous studies. Among the heretofore unstudied TGW, their equivalent HPV rates were comparable to GM. Current and investigational HPV vaccines could substantially protect GM, BM, and TGW from the serious consequences of HPV infection especially among HIV + MSM.

## Introduction

Human Papillomaviruses (HPV) are a group of DNA viruses commonly transmitted by sexual activity or other direct contact. They can infect the genital and anorectal areas, mouth, and throat of males and females.[1, 2] Over 40 types of HPV are reported, of which at least 13 are considered high-risk for leading to neoplasia in infected body sites. HPV infection is well-known for its association with the development of cervical cancer in women. To prevent this, HPV vaccination has been implemented for young women in many industrialized countries. [1, 3] There is also growing concern about the role of HPV in the increasing frequency of anal cancer in men who have sex with men (MSM) in developed countries. [4–6]

In Thailand, sex between men is common, with data from integrated analysis and advocacy across all surveys estimating that 2–15% of anatomical males have a history of sex with men. [7] There are many categories of gender roles, gender identities, sexual orientations, and behaviors within the broad classification referred to as MSM in Thailand. In general, they are comprised of three sub groups: gay men (GM) who self-identify as men and prefer insertive and/or receptive anal sex with other men, bisexual men (BM) who self-identify as men and engage in insertive and/or receptive anal sex with men and women, and transgender women (TGW) who are born as anatomical males (and who may or may not have undergone genital surgery), but who self-identify as women and prefer receptive anal sex with men.[8–10] These groups have different sexual practices that may pose different risks for acquisition of sexually transmitted infections (STIs), especially for HPV.

The prevalence of different infecting HPV types appears to vary both geographically and temporally. In 1990s, one HPV study among MSM in the USA reported the most common high-risk HPV types were 16, 18, 31 and 53 in descending frequency without any HPV vaccination available during that time. [11] A study of MSM in China reported in 2010 revealed that HPV type 16 was also the most frequent, followed by types 58 and 39. [12] In 2013, Phanuphak et al. also reported high rates of anal HPV infection of all types in MSM in Bangkok, Thailand, with frequencies among both HIV-positive (HIV+) of 85% and HIV-negative (HIV-) of 59%. [10] The most common high-risk HPV types were 16, 51 and 58. [10] These and most other similar studies were, however, in GM and/or BM subjects. [13–15] HPV types may also vary by risk group, but little is known about HPV frequency in TGW. Prior Thai studies focused on MSM in Bangkok or major tourist beach destinations such as Pattaya and Phuket. Equivalent data in northern Thailand has not been published. Right now in Thailand, an HPV vaccine is not included in the national immunization program either for boys or girls. Individuals who want to receive this vaccine need to pay for the whole course by themselves. The cost for this HPV vaccine in Thailand is around US $200–250 per person.

To provide a basis for future policymaking on the use of HPV vaccines in public immunization programs for high-risk individuals, we studied the prevalence of anal HPV, its infecting types, and risk factors for its acquisition among three subgroups of MSM in northern Thailand.

## Methods

### Study population and venue

The PIMAN Center is a clinic located in Chiang Mai city, northern Thailand, and has provided voluntary counseling and testing and STI treatment services for MSM since 2007. The clinic is operated by the Research Institute for Health Sciences (RIHES) of Chiang Mai University, and participates in a range of multi-site epidemiological and clinical studies in MSM populations, such as pre-exposure chemoprophylaxis for HIV prevention (e.g., the iPrEx trial) and rectal microbicides (e.g., the MTN-017 study). About 400 MSM clients who attended the PIMAN Center, between July 2012- January 2013, were approached about their interest in participating in this HPV study. The study inclusion criteria were self-identification as GM, BM, or TGW, age 18 years or older, and a history of anal receptive sexual intercourse in the preceding 6 months. Of those clients, there were 200 MSM who voluntarily provided informed consent to be enrolled in the study.

### Data and sample collection

Data were collected using a computer-assisted self-interview (CASI) program, and then by trained study staff in a private room. Each participant was assigned a unique identification code to anonymously link questionnaire responses and biologic specimens. Data collected via CASI or by staff included socio-demographic characteristics, substance use history, gender identity, history of HIV testing, history of prior STIs, and various sexual behaviors.

### HIV testing

The HIV status of study participants who reported being HIV+ from testing elsewhere were classified as such only if they provided written medical documentation of their status. Participants with undocumented, unknown, or prior HIV- status were provided with counseling and HIV testing, if consenting, to HIV testing; those declining HIV testing were classified as HIV-unknown (HIV^unk^).

HIV infection was determined by rapid test with the Alere Determine HIV 1/2 (Alere Medical Co, Ltd., Chiba, Japan), with confirmation of positives by enzyme-linked immunosorbent assay with the AxSYM HIV 1/2 gO (ABBOTT, Wiesbaden, Germany). For discordant results on these two tests, gelatin particle agglutination assay with the SERODIA—HIV (Fujirebio Inc., Tokyo, Japan) was used for final determination.

### Specimen collection

Anal samples for HPV DNA detection and genotyping were collected by physicians. A saline-moistened nylon swab was introduced into the anal canal to 5 cm depth. The swab was rotated in a circular motion for one minute, while gentle pressure was applied against the walls of the anal canal. The swab sample was placed immediately in thinPrep PreserveCyt solution (Hologic, Boxborough, MA, USA), and transported to the laboratory for cytology and HPV DNA testing.

### HPV detection and genotyping

DNA was extracted from the anal specimens using the DNeasy Blood & Tissue Kit (Qiagen, Hilden, Germany). Briefly, the cells were preserved in PreserveCyt solution and were pelleted by centrifugation at 1000 *g* at 20°C for 15 minutes. The cells were washed once with 1 x Dulbecco’s Phosphate-Buffered Saline (Invitrogen, Carlsbad, CA, USA), then subjected to DNA extraction according to the protocol provided by the manufacturer. The purified DNA was stored at -20°C until use.

Nested polymerase chain reaction (PCR) assays determined the presence of HPV in the 10 μl DNA extracts from the anal specimens, using consensus PGMY09/11/HMB01 HPV L1 primers in the first-round. These were followed by the GP5+/6+ primers in the second-round. [16] Co-amplification of the human β-globin gene in the first-round PCR was used as a positive control; samples that were negative for both HPV DNA and β-globin were considered inadequate, whereas those negative for GP5+/6+ and positive for β-globin were regarded as HPV DNA negative.

Samples that were positive for HPV using this approach were further analyzed using a line-blot assay (Linear Array HPV Genotyping Test [LA-HPV]; Roche Diagnostics GmBH, Mannheim, Germany), which detects 13 high-risk HPV genotypes (types 16, 18, 31, 33, 35, 39, 45, 51, 52, 56, 58, 59, and 68), and 24 low-risk genotypes (6, 11, 26, 40, 42, 53, 54, 55, 61, 62, 64, 66, 67, 69, 70, 71, 72, 73, 81, 82, 83, 84, IS39, and CP6108). Positive and negative controls used for the LA-HPV were those supplied with the kit. The probe for HPV type 52 is a mixed one that cross-reacts with types 33, 35, and 58; thus, identifications of type 52 reported here include only those without concurrent presence of the latter three types. Samples that tested positive by HPV DNA PCR but without a positive band on the LA-HPV strip were classified as type undetermined.

### Vaccine coverage

The HPV types identified were tabulated according to their coverage by the bivalent (16/18) and quadrivalent (16/18/6/11) commercially available vaccines, as well as by the 7-valent (16/18/31/33/45/52/58), and 9-valent (16/18/31/33/45/52/58/6/11) investigational ones. An HPV+ participant was considered “covered” by a vaccine if ≥1 of the type(s) identified to infect that participant was among a vaccine’s component antigens. Thus, our estimates are for “partial” coverage only, because a vaccine’s antigens may only correspond to some—but not all—of the HPV infections in a participant co-infected with multiple types.

### Statistical analysis

The data were analyzed statistically using Stata/IC for Windows, Version 10.0 (StataCorp LP, College Station, TX, USA). Statistical significance for categorical data was determined by Pearson’s Chi-square test. Paired proportions were compared by McNemar's test. For bivariate data, odds ratios (OR) and 95% confidence intervals (95% CI) were calculated and statistical significance determined by Chi-square (χ^2^) test.

Potential demographic, sexual, behavioral, and other risk factors or predictors for the assessed outcome of HPV infection were analyzed by multivariate logistic regression. The variable-selection process for the multivariate model began with bivariate analysis of each variable, for which those with p values of <0.25 were candidates for the multivariate analysis. When specific analytical data cells had counts of zero, exact logistic regression was used. Forward selection was then applied by starting with a simple model and adding terms sequentially until further additions did not significantly improve the fit. Covariates were removed from the model if non-significant and not confounders. [17]

### Ethics

Study protocols, questionnaires, and written informed consent were approved by the Ethics Committee of the Research Institute for Health Sciences, Chiang Mai University. PIMAN Center attendees were provided with information about the study and invited to participate. Only those who voluntarily provided written informed consent were enrolled. Participants were informed of their study test results for HIV and HPV, followed by appropriate counseling and treatment. Any STIs diagnosed in the course of the study were treated by the study team at the PIMAN Center, and/or referred to appropriate clinical facilities elsewhere for further treatment. Publication of the study data is in accordance with the community standards and approved by the ethics committee.

## Results

### Participant characteristics

The study population comprised equal numbers of GM (85, 42.5%) and TGW (85, 42.5%), while 30 (15%) BM constituted the remainder (Table 1). GM were twice as likely to have any university education (62%) than either BM (30%) or TGW (28%). The majority (55%) of the participants was between 20 and 29 years old (overall age range 18–54 years) and 43% had a university degree.

**Table 1:** Demographic characteristics of age, education, occupation, and nationality, by gender-identity of MSM subjects in Chiang Mai, Thailand, 2012–2013.

Demographic Variables	Gender identity			Totals	
	Gay Men (GM)	Bisexual Men (BM)	Transgender Women (TGW)		Statistic, *p-value*
	(N = 85)	(N = 30)	(N = 85)	(N = 200)	
**Age in years, N (%)**					
< 20	8 (9)	4 (13)	15 (18)	27 (14)	χ^2^_(6)_ = 12.6, ***p = 0*.*049***
20–29	40 (47)	19 (63)	51 (60)	110 (55)	
30–39	27 (32)	7 (23)	14 (16)	48 (24)	
≥ 40	10 (12)	0	5 (6)	15 (8)	
*Mean / Median*	*29*.*5 / 27*	*25*.*2 / 24*	*25*.*6 / 23*	*27*.*2 / 25*	
*Min*.*-Max*.	*18–54*	*18–36*	*18–48*	*18–54*	
**Highest education level, N(%)**					
None	3 (4)	4 (13)	5 (6)	12 (6)	χ^2^_(8)_ = 30.9, ***p = 0*.*000***
Any primary (1 to ≤6 grades)	0	1 (3)	0	1 (1)	
Any secondary (1 to ≤6 grades)	13 (15)	7 (23)	22 (26)	42 (21)	
Any vocational (after 2° grade 3)	16 (19)	9 (30)	34 (40)	59 (30)	
Any university	53 (62)	9 (30)	24 (28)	86 (43)	
**Main occupation, N(%)**					
Student	23 (27)	5 (17)	32 (38)	60 (30)	χ^2^_(4)_ = 15.8, ***p = 0*.*003***
Employed	58 (68)	17 (57)	43 (51)	118 (59)	
Unemployed	4 (5)	8 (27)	10 (12)	22 (11)	
**Nationality, N(%)**					
Thai	84 (99)	29 (97)	85 (100)	198 (99)	χ^2^_(2)_ = 2.5, *p = 0*.*280*, NS
Thai ethnic minority	1 (1)	1 (3)	0	2 (1)	

### Sexual behavior and history

Five variables of sexual behavior stood out for statistically-significant differences between the gender-identity groups (Table 2). TGW reported a higher frequency of exclusively receptive anal sex than both the GM and BM (p < 0.001). GM reported less-frequent sexual intercourse with men or women than the BM and TGW groups (p < 0.001). GM consumed alcohol before sex less frequently than BM and TGW (p < 0.01). BM received something of value in exchange for sex more often than GM or TGW (p < 0.0001). TGW reported lower frequency than GM and BM of penile symptoms suggestive of STIs (p <0.001).

**Table 2:** Frequency of sexual and other behaviors, by gender-identity of MSM subjects in Chiang Mai, Thailand, 2012–2013.

Sexual Behavior Variables	Gender identity			Totals	
	Gay Men (GM)	Bisexual Men (BM)	Transgender Women (TGW)		Statistic, *p-value*
	(N = 85)	(N = 30)	(N = 85)	(N = 200)	
**Regular partner status, N(%)**					χ^2^_(2)_ = 3.4, *p = 0*.*187*, NS
No	61 (72)	26 (87)	68 (80)	155 (78)	
Yes	24 (28)	4 (13)	17 (20)	45 (22)	
**Sexual role, N(%)**					χ^2^_(4)_ = 69.0, ***p = 0*.*000***
Insertive only	0	3 (10)	1 (1)	4 (2)	
Receptive only	40 (47)	5 (17)	76 (89)	121 (60)	
Both insertive and receptive	45 (53)	22 (73)	8 (9)	75 (38)	
**Last 6 months, anal intercourse with sex partner(s), N(%)**					
None	1 (1)	0	2 (2)	3 (2)	χ^2^_(6)_ = 4.8, *p = 0*.*310*, NS
Yes	78 (95)	24 (89)	80 (95)	182 (94)	
No sex at all	3 (4)	3 (11)	2 (2)	8 (4)	
Declined to answer *	3	3	1	7	
**If yes, use condom all the time last 6 months**					χ^2^_(2)_ = 4.5, *p = 0*.*107*, NS
Yes	38 (49)	7 (29)	28 (35)	73 (40)	
No	40 (51)	17 (71)	52 (65)	109 (60)	
**Sexual intercourse frequency with men or women, N(%)**					χ^2^_(6)_ = 26.9, ***p = 0*.*000***
Everyday	0	4 (13)	8 (9)	12 (6)	
3–4 times per week	12 (14)	12 (40)	23 (27)	47 (24)	
1–2 times per week	27 (32)	6 (20)	30 (35)	63 (32)	
One per month or less	46 (54)	8 (27)	24 (28)	78 (39)	
**Drink alcohol before sex with BM, GM, or TGW, N(%)**					χ^2^_(6)_ = 17.8, ***p = 0*.*007***
Every time	1 (1)	6 (20)	5 (6)	12 (6)	
Almost every time	8 (9)	4 (13)	13 (15)	25 (12)	
Sometimes	45 (53)	13 (43)	47 (55)	105 (52)	
Never	31 (36)	7 (23)	20 (24)	58 (29)	
**Ever give money, gifts or valuables in exchange for sex with BM, GM or TGW, N(%)**					χ^2^_(2)_ = 2.9, *p = 0*.*238*, NS
No	62 (73)	24 (80)	71 (84)	157 (78)	
Yes	23 (27)	6 (20)	14 (16)	43 (22)	
**If yes, when was the last time**					χ^2^_(2)_ = 2.1, *p = 0*.*354*, NS
1 year or less	17 (74)	5 (83)	13 (93)	35 (81)	
More than 1 year	6 (26)	1 (17)	1 (7)	8 (19)	
**Ever receive money, gifts or valuables in exchange for sex, N(%)**					χ^2^_(2)_ = 20.7, ***p = 0*.*000***
Never	71 (84)	12 (40)	60 (71)	143 (72)	
Ever	14 (16)	18 (60)	25 (29)	57 (28)	
**If ever last time**					χ^2^_(2)_ = 0.9, *p = 0*.*642*, NS
1 year or less	10 (71)	15 (83)	18 (72)	43 (75)	
More than 1 year	4 (29)	3 (17)	7 (28)	14 (25)	
**Ever circumcised?, N(%)**					χ^2^_(2)_ = 0.7, *p = 0*.*705*, NS
Yes	4 (5)	1 (3)	2 (2)	7 (4)	
No	81 (95)	29 (97)	83 (98)	193 (96)	
**Number of total sex partner(s) last 6 months, N(%)**					χ^2^_(6)_ = 11.0, *p = 0*.*088*, NS
None	3 (4)	3 (11)	2 (2)	8 (4)	
1	20 (24)	3 (11)	19 (23)	42 (22)	
2–4	34 (41)	6 (22)	31 (37)	71 (37)	
5 or more	25 (30)	15 (56)	32 (38)	72 (37)	
Declined to answer *	3	3	1	7	
*Mean / Median*	*4*.*6 / 3*	*14*.*2 / 7*	*10*.*8 / 3*.*5*	*8*.*6 / 3*	
*Min*.*-Max*.	*0–45*	*0–103*	*0–300*	*0–300*	
**Ever have unusual fluid, urinary irritation, ulcer, or rash on penis in last 6 months, N(%)**					χ^2^_(2)_ = 16.1, ***p = 0*.*000***
Yes	17 (20)	9 (30)	3 (4)	29 (14)	
No	68 (80)	21 (70)	82 (96)	171 (86)	
**If yes, see doctor and get diagnosis**					χ^2^_(2)_ = 1.4, *p = > 0*.*507*, NS
Yes	9 (53)	3 (33)	2 (67)	14 (48)	
No	8 (47)	6 (67)	1 (33)	15 (52)	

* Not included in testing for statistical significance.

### HIV and HPV prevalence and co-infections

Of the 200 participants, 49 (24.5%) were HIV+, 122 (61.0%) HIV-, and 29 (14.5%) HIV^unk^ (Table 3). HPV infection with any type was found in 157 (79.7%) of 197 with adequate anal-swab samples. Among the HIV+ subjects, all 48 (100%) with adequate specimens were found on Linear Array HPV assay to be infected with one or more HPV types, which was significantly higher than the 70% rate (84 of 120) among HIV- subjects (*p* <0.01).

**Table 3:** HPV infections and co-infections by HIV infection status of MSM subjects studied in Chiang Mai, Thailand, 2012–2013.

Variables	HIV Infection			Totals	
	HIV+	HIV-	HIV^unk^ *		Statistic, *p-value*
	(N = 49)	(N = 122)	(N = 29)	(N = 200)	
**HPV DNA by PCR, N(%)**					χ^2^_(1)_ = 12.3, ***p = 0*.*000***
HPV+	48 (100)	94 (78)	25 (86)	167 (85)	
HPV-	0	26 (22)	4 (14)	30 (15)	
Inadequate specimen*	1	2	0	3	
**HPV DNA by linear array, N(%)**					χ^2^_(1)_ = 15.0, ***p = 0*.*000***
HPV+	48 (100)	84 (74)	25 (86)	157 (83)	
HPV-	0	29 (26)	4 (14)	33 (17)	
Undetermined type, Not specified*	0	7	0	7	
**Number HPV co-infection, N(%)**					χ^2^_(5)_ = 32.2, ***p = 0*.*000***
None	0	36 (30)	4 (14)	40 (20)	
1	2 (4)	20 (17)	6 (21)	28 (14)	
2	5 (10)	15 (12)	5 (17)	25 (13)	
3–5	25 (52)	34 (28)	5 (17)	64 (32)	
6–8	14 (29)	13 (11)	7 (24)	34 (17)	
≥ 9	2 (4)	2 (2)	2 (7)	6 (3)	
*Mean / Median*	*4*.*8 / 4*	*3*.*4 / 3*	*4*.*1 / 3*	*3*.*9 / 4*	
*Min*.*-Max*.	*1–12*	*1–10*	*1–9*	*1–12*	
**Frequency of low and high risk HPV types, N(%)**					χ^2^_(3)_ = 25.0, ***p = 0*.*000***
None	0	36 (30)	4 (14)	40 (20)	
Only low risk(LR) type(s)	3 (6)	19 (16)	3 (10)	25 (13)	
Only high risk(HR) type(s)	5 (10)	8 (7)	4 (14)	17 (9)	
Both LR and HR type(s)	40 (83)	57 (48)	18 (62)	115 (58)	
**Number of high-risk HPV co-infection(s), N(%)**					χ^2^_(4)_ = 28.8, ***p = 0*.*000***
None	3 (6)	55 (46)	7 (24)	65 (33)	
1	11 (23)	20 (17)	12 (41)	43 (22)	
2	12 (25)	22 (18)	1 (3)	35 (18)	
3–5	18 (38)	22 (18)	8 (28)	48 (24)	
6–8	4 (8)	1 (1)	1 (3)	6 (3)	
*Mean / Median*	*2*.*8 / 2*	*2*.*2 / 2*	*2*.*2 / 1*	*2*.*4 / 2*	
*Min*.*-Max*.	*1–7*	*1–7*	*1–6*	*1–7*	

* Not included in testing for statistical significance.

Infection with multiple HPV types was common (Table 3): 13% (25/197) were co-infected with two types, 32% (64) 3-to-5 types, 17% (37) 6-to-8 different types, and 3% (6) ≥9 types. The mean number of HPV types was 4.8 among HIV+ participants (range 1 to 12), compared to 3.4 (range 1–10) in the HIV- (*p* < 0.001)

HPV infection with any of the 13 high-risk types was found in 132 of 197 (67.0%) (Table 3). Co-infections with ≥1 high-risk types among HIV+ was 94% (45 of 48), and in HIV- 54% (65 of 120) (*p <* 0.0001).

### HPV types and vaccine coverage

**Fig 1:**
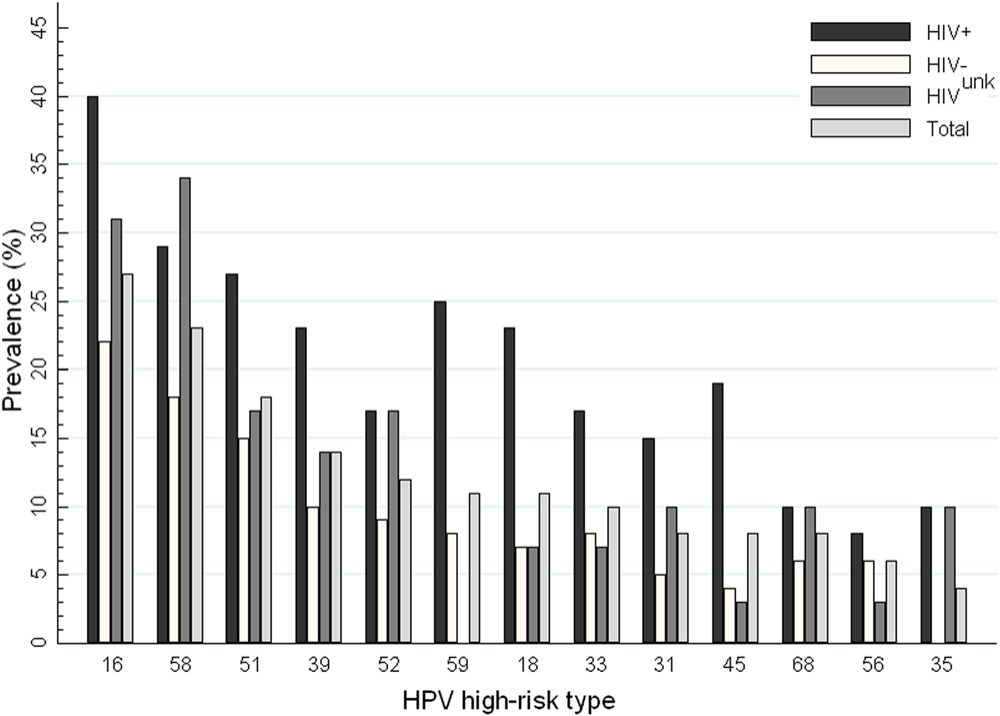
Prevalence of high-risk HPV types, by HIV status, ranked from left to right by decreasing percentage among total participants.

The most common high-risk HPV types were 16 (27%), 58 (23%), 51(18%), and 39 (14%) (Fig 1). Infection by type 16 was more common in HIV+ (40%, 19/48), versus 22% (26/120) in HIV- (*p* < 0.02). Increasing vaccine valences from 2, to 4, to 7, and to 9 increased the percentage of participants who were infected with at least one HPV type covered in a vaccine from 35.5% (70/197), to 48.2% (95), to 56.4% (111), and to 64.0% (126), respectively (Table 4). HPV vaccine coverage for either HIV+ or HIV- increased steadily as valency increased from 2 to 4, to 7, and to 9 (Table 5).

**Table 4:** Coverage by commercial^a^,^b^ and investigational^c^,^d^ HPV vaccines of ≥1 HPV infecting type(s), by gender identity of MSM subjects in Chiang Mai, Thailand, 2012–2013.

	No. of participant / Total cases (%)	Increased percentage coverage above 2-valent (95% CI)	Ratio to 2-valent (95% CI)	Statistic, *p-value*
**Gay Men, N(%)**				
2-valent	33 / 85 (38.8)	Ref.		
4-valent	45 / 85 (52.9)	+14.1 (5.5–22.7)	1.4 (1.1–1.6)	χ^2^_(1)_ = 12.0, ***p = 0*.*0005***
7-valent	54 / 85 (63.5)	+24.7 (14.4–35.1)	1.6 (1.3–2.0)	χ^2^_(1)_ = 21.0, ***p = 0*.*000***
9-valent	61 / 85 (71.8)	+32.9 (21.8–44.1)	1.9 (1.5–2.3)	χ^2^_(1)_ = 28.0, ***p = 0*.*000***
**Bisexual Men, N(%)**				
2-valent	7 / 29 (24.1)	Ref.		
4-valent	10 / 29 (34.5)	+10.3 (-4.2–24.9)	1.4 (1.0–2.1)	χ^2^_(1)_ = 3.0, *p = 0*.*083*, *NS*
7-valent	8 / 29 (27.6)	+3.5 (-6.6–13.5)	1.1 (0.9–1.5)	χ^2^_(1)_ = 1.0, *p = 0*.*317*, *NS*
9-valent	11 / 29 (37.9)	+13.8 (-2.2–29.8)	1.6 (1.0–2.5)	χ^2^_(1)_ = 4.0, ***p = 0*.*046***
**Transgender Women, N(%)**				
2-valent	30 / 83 (36.1)	Ref.		
4-valent	40 / 83 (48.2)	+12.1 (3.8–20.3)	1.3 (1.1–1.6)	χ^2^_(1)_ = 10.0, ***p = 0*.*002***
7-valent	49 / 83 (59.0)	+22.9 (12.7–33.1)	1.6 (1.3–2.0)	χ^2^_(1)_ = 19.0, ***p = 0*.*000***
9-valent	54 / 83 (65.1)	+28.9 (18.0–39.9)	1.8 (1.4–2.3)	χ^2^_(1)_ = 24.0, ***p = 0*.*000***
**All participant, N(%)**				
2-valent	70 / 197 (35.5)	Ref.		
4-valent	95 / 197 (48.2)	+12.7 (7.5–17.8)	1.4 (1.2–1.5)	χ^2^_(1)_ = 25.0, ***p = 0*.*000***
7-valent	111 / 197 (56.4)	+20.8 (14.6–27.0)	1.6 (1.4–1.8)	χ^2^_(1)_ = 41.0, ***p = 0*.*000***
9-valent	126 / 197 (64.0)	+28.4 (21.6–35.2)	1.8 (1.5–2.1)	χ^2^_(1)_ = 56.0, ***p = 0*.*000***

^***a***^ 2–valent coverage: number infected with ≥1 HPV type(s) 16 and/or 18

^***b***^ 4–valent coverage: number infected with ≥1 HPV type(s) 16, 18, 6, and/or 11

^***c***^ 7–valent coverage: number infected with ≥1 HPV type(s) 16, 18, 31, 33, 45, 52, and/or 58

^***d***^ 9–valent coverage: number infected with ≥1 HPV type(s) 16, 18, 31, 33, 45, 52, 58, 6, and/or 58).

**Table 5:** Coverage by commercial^a^,^b^ and investigational^c^,^d^ HPV vaccines of ≥1 HPV infecting type(s), by HIV status of MSM subjects in Chiang Mai, Thailand, 2012–2013.

	No. of participant / Total cases (%)	Increased percentage coverage above 2-valent (95% CI)	Ratio to 2-valent (95% CI)	Statistic, *p-value*
**HIV+, N(%)**				
2-valent	28 / 48 (58.3)	Ref.		
4-valent	34 / 48 (70.8)	+12.5 (1.1–23.9)	1.2 (1.0–1.4)	χ^2^_(1)_ = 6.0, ***p = 0*.*014***
7-valent	39 / 48 (81.3)	+22.9 (8.9–36.9)	1.4 (1.1–1.7)	χ^2^_(1)_ = 11.0, ***p = 0*.*001***
9-valent	42 / 48 (87.5)	+29.2 (14.2–44.1)	1.5 (1.2–1.9)	χ^2^_(1)_ = 14.0, ***p = 0*.*0002***
**HIV-, N(%)**				
2-valent	32 / 120 (26.7)	Ref.		
4-valent	47 / 120 (39.2)	+12.5 (5.8–19.3)	1.5 (1.2–1.8)	χ^2^_(1)_ = 15.0 ***p = 0*.*0001***
7-valent	55 / 120 (45.8)	+19.2 (11.3–27.0)	1.7 (1.4–2.2)	χ^2^_(1)_ = 23.0, ***p = 0*.*000***
9-valent	65 / 120 (54.2)	+27.5 (18.7–36.3)	2.0 (1.6–2.6)	χ^2^_(1)_ = 33.0, ***p = 0*.*000***
**HIV** ^**unk**^, **N(%)**				
2-valent	10 / 29 (34.5)	Ref.		
4-valent	14 / 29 (48.3)	+13.8 (-2.2–29.8)	1.4 (1.1–2.0)	χ^2^_(1)_ = 4.0, ***p = 0*.*046***
7-valent	17 / 29 (58.6)	+24.1 (5.1–43.2)	1.7 (1.1–2.5)	χ^2^_(1)_ = 7.0, ***p = 0*.*008***
9-valent	19 / 29 (65.5)	+31.0 (10.8–51.3)	1.9 (1.2–2.9)	χ^2^_(1)_ = 9.0, ***p = 0*.*003***
**All participant, N(%)**				
2-valent	70 / 197 (35.5)	Ref.		
4-valent	95 / 197 (48.2)	+12.7 (7.5–17.8)	1.4 (1.2–1.5)	χ^2^_(1)_ = 25.0, ***p = 0*.*000***
7-valent	111 / 197 (56.4)	+20.8 (14.6–27.0)	1.6 (1.4–1.8)	χ^2^_(1)_ = 41.0, ***p = 0*.*000***
9-valent	126 / 197 (64.0)	+28.4 (21.6–35.2)	1.8 (1.5–2.1)	χ^2^_(1)_ = 56.0, ***p = 0*.*000***

^***a***^ 2–valent coverage: number infected with ≥1 HPV type(s) 16 and/or 18

^***b***^ 4–valent coverage: number infected with ≥1 HPV type(s) 16, 18, 6, and/or 11

^***c***^ 7–valent coverage: number infected with ≥1 HPV type(s) 16, 18, 31, 33, 45, 52, and/or 58

^***d***^ 9–valent coverage: number infected with ≥1 HPV type(s) 16, 18, 31, 33, 45, 52, 58, 6, and/or 58.

### Determinants of anal HPV infection

On univariate analysis, the odds ratio (OR) for anal HPV infection with any type virus among GM was 9.0 (95% CI 2.97–28.03, *p* <0.001), and among TGW 4.5 (95% CI 1.63–12.26, *p* = 0.001), compared to BM as the referents (Table 6). The adjusted odd ratio (AOR) by exact logistic regression for GM was 5.3 (95% CI 1.2–25.7, *p* = 0.027), compared to BM as referents.

**Table 6:** Crude and adjusted Odds ratios by bivariate and exact logistic regression for any HPV positivity by demographic, behavioral, and sexual risk factors of MSM in Chiang Mai, Thailand.

Characteristics	No. cases	Bivariate analysis				Exact logistic regression analysis ^a^		
		No. HPV+ (%)	OR	95% CI	*p-value*	AOR ^b^	95% CI	*p-value*
**Gender identity**								
Bisexual men	29	14 (48)	*Ref*.			*Ref*.		
Gay men	85	76 (89)	9.0	3.0–28.0	***0*.*000***	5.3	1.2–25.7	***0*.*027***
Transgender women	83	67 (81)	4.5	1.6–12.3	***0*.*001***	4.0	1.0–16.8	*0*.*055*
**HIV status**								
Negative	120	84 (70)	*Ref*.			*Ref*.		
Positive	48	48 (100)	41.9	2.5–698.0	***0*.*001***	20.3	3.3 - +inf.	***0*.*0002***
Unknown	29	25 (86)	2.7	0.8–11.3	*0*.*078*	3.3	0.9–16.9	*0*.*080*
**Age in years**								
< 20	27	22 (82)	*Ref*.					
20–29	108	84 (78)	0.8	0.2–2.5	*0*.*675*			
30–39	47	37 (79)	0.8	0.2–3.2	*0*.*776*			
40 or more	15	14 (93)	3.2	0.3–161.0	*0*.*293*			
**Sexual role**								
Insertive only & both	78	58 (74)	*Ref*.					
Receptive only	119	99 (83)	1.7	0.8–3.6	*0*.*132*			
**Ever used hormones**								
Never	98	76 (78)	*Ref*.					
Ever	99	81 (82)	1.3	0.6–2.8	*0*.*457*			
**Alcohol consumption**								
Never	18	15 (83)	*Ref*.					
Ever	179	142 (79)	0.8	0.1–2.9	*0*.*687*			
**Frequency of sexual intercourse with BM, GM or TGM**								
1–2 times/week or less	140	111 (79)	*Ref*.					
3–4 times/week or more	57	46 (81)	1.1	0.5–2.6	*0*.*823*			
**Drank alcohol before having sex with BM, GM or TGW**								
Never / sometime	162	135 (83)	*Ref*.			*Ref*.		
Almost every time / Every time	35	22 (63)	0.3	0.1–0.8	***0*.*006***	0.4	0.1–1.3	*0*.*127*
**BM, GM or TGW partner drank alcohol before having sex with you**								
Never / sometime	164	134 (82)	*Ref*.					
Almost every time/ Every time	33	23 (70)	0.5	0.2–1.4	*0*.*118*			
**Ever received money, gifts or valuables in exchange for sex**								
Never	141	114 (81)	*Ref*.					
Ever	56	43 (77)	0.8	0.4–1.8	*0*.*522*			
**Ever had any fluid, irritating urinary symptom, ulcer, or rash on your penis**								
No	169	135 (80)	*Ref*.					
Yes	28	22 (79)	0.9	0.3–3.0	*0*.*873*			
**Total sex partner(s) last 6 months**								
0–1	49	35 (71)	*Ref*.			*Ref*.		
2 or more	141	117 (83)	2.0	0.8–4.4	*0*.*082*	2.2	0.8–6.0	*0*.*157*
**Number of female sex partner(s) last 6 months**								
0–1	172	143 (83)	*Ref*.			*Ref*.		
2 or more	16	7 (44)	0.2	0–0.5	***0*.*000***	0.3	0.0–2.4	*0*.*368*
**Use condom all the time last 6 months with sex partner(s)**								
No sex partner / No anal sex	10	7 (70)	*Ref*.					
Yes	73	60 (82)	2.0	0.3–10.1	*0*.*359*			
No	107	85 (79)	1.7	0.2–8.0	*0*.*486*			
**Use condom all the time last 6 months with** **regular** **sex partner(s)**								
No sex partner / No anal sex	74	56 (76)	*Ref*.					
Yes	46	38 (83)	1.5	0.6–4.5	*0*.*370*			
No	73	59 (81)	1.4	0.6–3.2	*0*.*450*			
**Use condom all the time last 6 months with** **casual** **sex partner(s)**								
No sex partner / No anal sex	47	36 (77)	*Ref*.					
Yes	75	61 (81)	1.3	0.5–3.5	*0*.*528*			
No	72	59 (82)	1.4	0.5–3.8	*0*.*477*			

^**a**^ Exact logistic regression used for these estimates due to zero cell counts

^**b**^ AOR = adjusted odd ratio. All measures adjusted for drinking alcohol before having sex, number of total sex partner(s) in last 6 months and number of female sex partner(s) in last 6 months

OR = crude odds ratio

Inf. = infinity

*Ref*. = Reference value.

For HIV+ participants, the any-type bivariate OR was 41.9 (95% CI 2.54–698.02, *p* = 0.001), compared to the HIV- as the referent. The AOR for the HIV+ was 20.29 (95% CI 3.28-∞, *p* < 0.001), compared to the HIV-.

Infection with any of the 13 high-risk HPV subtypes showed a bivariate OR of 5.4 for GM (95% CI 2.01–15.03, *p* <0.001) and 4.67 for TGW (95% CI 1.74–12.85, *p* <0.001), compared to BM referents. The multivariate AOR for GM was 4.2 (95% CI 1.2–14.6, *p* = 0.021) and for TGW was 6.2 (95% CI 1.8–20.9, *p* = 0.003), compared to BM (Table 7).

**Table 7:** Crude and adjusted odds ratios by bivariate and logistic regression, for high-risk-type HPV positivity, by demographic, behavioral, and sexual risk factors of MSM in Chiang Mai, Thailand.

Characteristics	No. cases	Bivariate analysis				Logistic regression analysis		
		No. high risk HPV (%)	OR	95% CI	*p-value*	AOR ^a^	95% CI	*p-value*
**Gender identity**								
Bisexual men	29	10 (35)	*Ref*.			*Ref*.		
Gay men	85	63 (74)	5.4	2.0–15.0	***0*.*000***	4.2	1.2–14.6	***0*.*021***
Transgender women	83	59 (71)	4.7	1.7–12.8	***0*.*000***	6.2	1.8–20.9	***0*.*003***
**HIV status**								
Negative	120	65 (54)	*Ref*.			*Ref*.		
Positive	48	45 (94)	12.7	3.7–66.5	***0*.*000***	12.8	3.6–45.8	***0*.*000***
Unknown	29	22 (76)	2.7	1.0–7.9	***0*.*033***	3.1	1.1–8.6	***0*.*033***
**Age in years**								
< 20	27	20 (74)	*Ref*.					
20–29	108	67 (62)	0.6	0.2–1.6	*0*.*243*			
30–39	47	32 (68)	0.8	0.2–2.4	*0*.*587*			
40 or more	15	13 (87)	2.3	0.4–25.3	*0*.*341*			
**Sexual role**								
Insertive only & both	78	47 (60)	*Ref*.					
Receptive only	119	85 (71)	1.6	0.9–3.2	*0*.*103*			
**Ever used hormones**								
Never	98	61 (62)	*Ref*.					
Ever	99	71 (72)	1.5	0.8–2.9	*0*.*157*			
**Alcohol consumption**								
Never	18	14 (78)	*Ref*.					
Ever	179	118 (66)	0.6	0.1–1.9	*0*.*308*			
**Frequency of sexual intercourse with BM, GM or TGM**								
1–2 times/week or less	140	96 (69)	*Ref*.					
3–4 times/week or more	57	36 (63)	0.8	0.4–1.6	*0*.*464*			
**Drank alcohol before having sex with BM, GM or TGW**								
Never / sometime	162	113 (70)	*Ref*.			*Ref*.		
Almost every time / Every time	35	19 (54)	0.5	0.2–1.2	*0*.*078*	0.6	0.2–1.4	*0*.*223*
**Your BM, GM or TGW partner drank alcohol before having sex with you**								
Never / sometime	164	111 (68)	*Ref*.					
Almost every time / Every time	33	21 (64)	0.8	0.4–2.0	*0*.*652*			
**Ever received money, gifts or valuables in exchange for sex**								
Never	141	99 (70)	*Ref*.					
Ever	56	33 (59)	0.6	0.3–1.2	*0*.*129*			
**Ever had any fluid, irritating urinary symptom, ulcer, or rash on your penis**								
No	169	111 (66)	*Ref*.					
Yes	28	21 (75)	1.6	0.6–4.6	*0*.*331*			
**Total sex partner(s) last 6 months**								
0–1	49	30 (61)	*Ref*.					
2 or more	141	97 (69)	1.4	0.7–2.9	*0*.*332*			
**Number of female sex partner(s) last 6 months**								
0–1	172	120 (70)	*Ref*.			*Ref*.		
2 or more	16	6 (38)	0.3	0.1–0.8	***0*.*009***	0.8	0.2–3.7	*0*.*798*
**Use condom all the time last 6 months with sex partner(s)**								
No sex partner / No anal sex	10	6 (60)	*Ref*.					
Yes	73	52 (71)	1.6	0.3–7.8	*0*.*468*			
No	107	69 (65)	1.2	0.2–5.5	*0*.*777*			
**Use condom all the time last 6 months with** **regular** **sex partner(s)**								
No sex partner / No anal sex	74	52 (70)	*Ref*.					
Yes	46	30 (65)	0.8	0.3–1.9	*0*.*563*			
No	73	46 (63)	0.7	0.3–1.5	*0*.*351*			
**Use condom all the time last 6 months with** **casual** **sex partner(s)**								
No sex partner / No anal sex	47	29 (62)	*Ref*.					
Yes	75	55 (73)	1.7	0.7–4.0	*0*.*177*			
No	72	47 (65)	1.2	0.5–2.7	*0*.*691*			

^**a**^ AOR = adjusted odd ratio. All measures adjusted for drinking alcohol before having sex and number of female sex partner(s) in last 6 months

OR = crude odds ratio

*Ref*. = Reference value.

For high-risk HPV subtypes, the HIV+ OR was 12.7 (95% CI 3.7–66.5, *p* < 0.001) compared to HIV-. The high-risk AOR for HIV+ was 12.8 (95% CI 3.6–45.8, *p* < 0.0001), compared to HIV-.

## Discussion

Our study showed an overall high prevalence of anal HPV infection with any of the 37 virus types assayed among northern Thai MSM who identified themselves as GM, BM, and TGW; and those who were HIV+ were 100% infected with HPV, significantly higher than those who were HIV- where the rate was 70%. This prevalence was higher than the 85% for HIV+ and 58.5% for HIV- MSM found in Bangkok in Central Thailand (nearly all were GM or BM), [10] as well as ever reported in other countries such as Brazil (65.6% for HIV+), [13] Taiwan (74.2% for HIV+), [14] and China (62–82% for HIV+, 58% for HIV-). [12,15]

For the 13 HPV subtypes considered high risk for disease sequelae, our infection prevalence was also higher among HIV+ and HIV- participants (94% and 54%, respectively), compared to HIV+ and HIV- MSM from Bangkok, 56.5% and 36.6%, respectively [10] (this prior study reported overall MSM rates). Our most common high risk subtype found in HIV+ MSM was HPV16 followed by 58, 51, and 39, findings quite similar to Bangkok MSM, where the most common of all subtypes were HPV16 (22.5%), 68 (13.3%), 58 (10.8%), 51 (10.8%) and 39 (10%). [10] Our prevalence of high-risk HPV was also higher than reported from Brazil (40.7% for HIV+), [13] Taiwan (40.4% for HIV+), [14] and China (61% for HIV+, 40% for HIV-). [15]

Self-reported gender identity of GM and TGW was independently associated with prevalent HPV infection, when compared with BM, particularly for infection with high-risk HPV types. The prevalence was highest in those who self-identified as GM (74%) and TGW (71%) compared with those who self-identified as BM (35%). Others in Thailand and elsewhere described the prevalence of HPV infection in MSM, [10–15, 18, 19] but did not examine HPV epidemiology in gender-identity subpopulations that differ in sexual behavior practices. In particular, TGW were absent from published studies in Thai MSM. [18] We are unaware of others reporting our high overall and high-risk type HPV rates in TGW. Our study identified and confirmed previous findings that HIV+ is a stronger risk factor for overall and high-risk HPV infections than HIV- and HIV^unk^ status. [12]

The difference of vaccine coverage and ratio (above 2-valent) of the 9-valent HPV vaccine of any HIV statuses or gendered groups were higher than other vaccines. Although the 2-valent, 4-valent, and 7-valent HPV vaccines had lower coverage than the 9-valent, as would be expected, nevertheless these lesser-valent vaccines still had high coverage in the subjects we studied.

For HIV- participants, unprotected receptive anal intercourse was not associated with HPV, as was reported from Italy, [19] while, in contrast, this was significantly associated with HPV infection in China. [15] Our lack of multivariate association of HPV with increased numbers of sex partners differed from data in Italy in which both lifetime and recent numbers of sexual partners were significantly associated with anal HPV infection. [19] Drinking alcohol before sex also lacked any multivariate association among our participants. In contrast, the HIM study in São Paulo (Brazil), Cuernavaca (Mexico), and Tampa (USA) demonstrated alcoholic drinking in the past month was significantly associated with oncogenic HPV infection. [20, 21]

Our study had several advantages in studying anal HPV prevalence, including a large number of MSM who identify themselves specifically as GM, BM, and TGW. Little is known about HPV infection in TGW and its risk factors. Computer-assisted interviewing with CASI (see methods) may have reduced response bias as homosexual sex is still stigmatized in Thailand.

There were a number of limitations. The cross-sectional design did not allow evaluation of HPV incidence, causation, and progression, and may have been affected by confounding. The number of participants with and without confirmed HIV infection was small because of the unknown HIV status of many participants, which limited statistical sub-analyses. The limited number of HIV+ participants precluded evaluation of CD4 count and antiretroviral drug use, which have been found to be significantly associated with HPV infection in some studies. [22, 23] Our findings of vaccine coverage were only “partial”, based on infection with one or more HPV types included in commercial and investigational vaccines. An analysis of “full” coverage of vaccines that would have prevented all the subtypes in patients infected with multiple types would have resulted in far lower coverage rates.

Our findings of HPV prevalence and vaccine coverage indicate that MSM in northern Thailand would benefit by protection from the serious consequences of HPV infection resulting from use of any of the HPV vaccines, with increasing protection as valences increases from 2 to 4 among commercial products, and from 7 to 9 among investigational ones. Further studies with follow-up of these and new consenting participants could evaluate in larger numbers the progression of HPV infection and its consequences.

## Supporting Information

S1 Dataset (XLS)
